# The First Genomic Study in a Romanian Spotted Calf with Ectopia Cordis Thoracalis

**DOI:** 10.3390/ijms27188179

**Published:** 2026-09-14

**Authors:** Viorel Herman, Simona Marc, Oana Maria Boldura, Alina Cărunta, Anca-Alexandra Tămaș, Ioan Claudiu Crăciun, Adrian Olariu-Jurca, Gabriel Otavă, Corina Badea, Alexandru Eugeniu Mizeranschi

**Affiliations:** 1 Faculty of Veterinary Medicine, University of Life Sciences ‘’King Mihai I’’ from Timisoara, Calea Aradului 119, 300645 Timisoara, Romania; viorel.herman@fmvt.ro (V.H.); oanaboldura@usvt.ro (O.M.B.); anca.tamas.iosud@usvt.ro (A.-A.T.); ioan.craciun@usvt.ro (I.C.C.); adrian.olariu-jurca@usvt.ro (A.O.-J.); gabrielotava@usvt.ro (G.O.); corina.badea@usvt.ro (C.B.); 2 Faculty of Informatics, West University from Timisoara, 4 Vasile Pârvan Blvd., 300223 Timișoara, Romania; alinacarunta@yahoo.com; 3 The Molecular Research Department, Research and Development Station for Bovine Arad, Bodrogului Street, 32, 310059 Arad, Romania

**Keywords:** cattle, ectopia cordis, renal agenesis, genomic characterization

## Abstract

Congenital heart defects (CHDs) are defects present at birth that can often lead to perinatal death, or, rarely, can be asymptomatic and detected later in life. Ectopia cordis is a rare type of malformation in which the heart is not located in its normal position. Other congenital abnormalities can be associated with this condition. In this article, we describe a case of ectopia cordis thoracalis with atrial septal defect in association with unilateral renal agenesis in a male Romanian Spotted calf. Clinical examination, dissection, and whole-genome sequencing were performed to describe and identify a possible cause of the abnormalities. Clinical examination revealed the presence of the heart in the thoracic region, subcutaneously, without apparent impairment of major physiological functions. Upon dissection, the external appearance of the heart was normal. However, the internal examination revealed an atrial septal defect measuring 1 cm in diameter in the proximal third of the interatrial septum. The case presented unilateral agenesis of the left kidney. VEP annotation of the joint callset identified 2270 missense variant annotations predicted as deleterious by SIFT and, separately, 12 high-impact start-loss variants by VEP consequence annotation. The *OR51F5C* and *OR8J17* variants were homozygous in the calf. Analyzing genes involved in cardiac and renal development, the following genes—*SHH*, *TBX18*, *NOTCH1*, *GATA3*, *TBX2*, *FOXC1*, and *RET* had mutations with moderate impact. Mutations in the *FOXC1* gene occurred in homozygous states for both the calf and mother genomes, while the *RET* gene had two mutations in a homozygous state just in the calf genome, and in a heterozygous state in the mother’s genome. One limitation of the present study is the unavailability of the paternal genome for comparative analysis; in addition, maternal genomic profiling did not reveal a contribution to the identified variants. Although the specific pathogenic relevance of the variants detected to the observed phenotype cannot be conclusively established, the findings expand the current genomic knowledge of this rare disorder and provide valuable data for future investigations into its genetic basis.

## 1. Introduction

Ectopia cordis (EC), or ectopic heart, is a rare malformation characterized by the atypical position of the heart outside the thoracic cavity [1]. EC in humans was first described in 1706 by Haller [2,3]. The prevalence in human medicine is estimated at 5.5–7.9/1,000,000 births, being more common in female fetuses and accounting for 0.1% of congenital heart diseases [1,4]. In veterinary medicine, ectopia cordis has been reported in cattle [5,6], sheep [7], goats [8,9], pigs [10], dogs [11] and cats [12]. Based on the anatomical region in which the heart is located, there are several types: cervical (superior and inferior), thoracic (sternal), thoraco-abdominal and abdominal [5,6,13]. According to Jezek et al. [5], the inferior cervical type is the most reported type of ectopia cordis, while according to Ghisu et al. [4], the thoracic type is the most common type. In the thoracic type, the heart is partially or completely displaced due to a defect in the sternum or ribs. The abdominal type is the least frequent.

Until 2011, 118 cases were reported in the specialized literature in cattle, of which 82% were cervical, 14% were thoracic, and 3% were abdominal [5,6]. The presence of other defects is a common occurrence with an ectopic heart, such as valve disorders, anencephaly, hydrocephalus, pulmonary hypoplasia, genitourinary malformations, cleft lip and palate, or gastrointestinal defects, which reduce the animal’s lifespan [14]. The mortality risk in this pathology depends on whether the evolution is syndromic or not, as well as whether there are other cardiac anomalies. In most documented cases, the clinical presentation is syndromic and frequently accompanied by complex cardiac defects. Consequently, the reported lifespan in bovine cases of ectopia cordis exhibits substantial variability, ranging from a few minutes postpartum to several years [5,6]. One example is a Holstein female with cervical ectopic heart, associated with dysplasia of the tricuspid valve, which not only survived with these birth defects, but also calved at 3 years old without periparturient problems [6]. Breeds in which cases of ectopic heart have been reported are Holstein Friesian, Angus, Shorthorn, Hereford, Guernsey, Simmental, Limousin and mixed breed [5].

The aim of the study is to describe EC thoracalis in a male Romanian Spotted calf from the northwestern region of Romania and to explore whether whole-genome sequencing can identify candidate variants in genes relevant to cardiac, thoracic wall, or renal development, including variants shared with or absent from the dam.

## 2. Results

### 2.1. Clinical Findings

**Figure 1:**
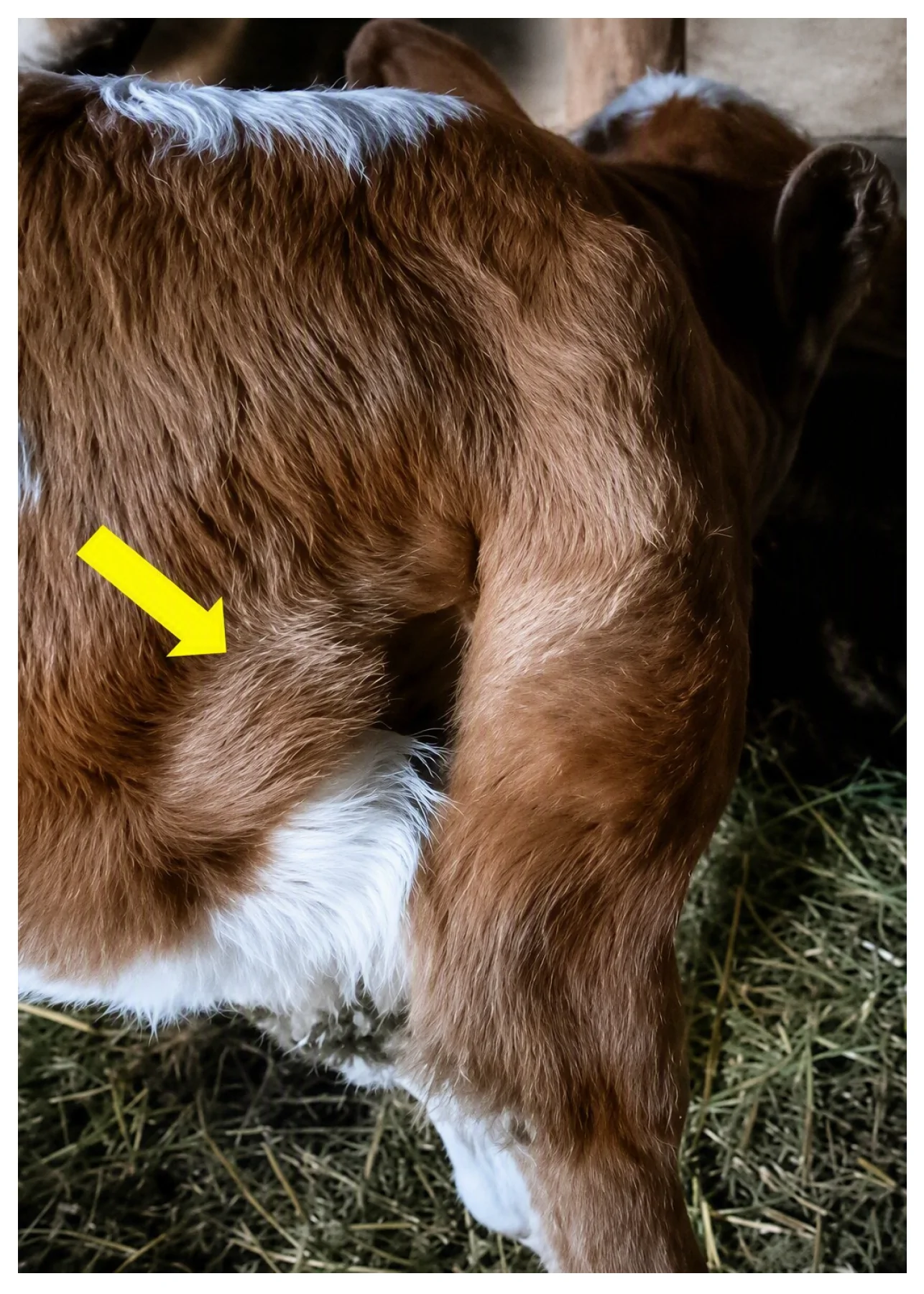
Clinical aspects of the calf: right thoracic view of the calf with the subcutaneous ectopic heart (yellow arrow).

**Figure 2:**
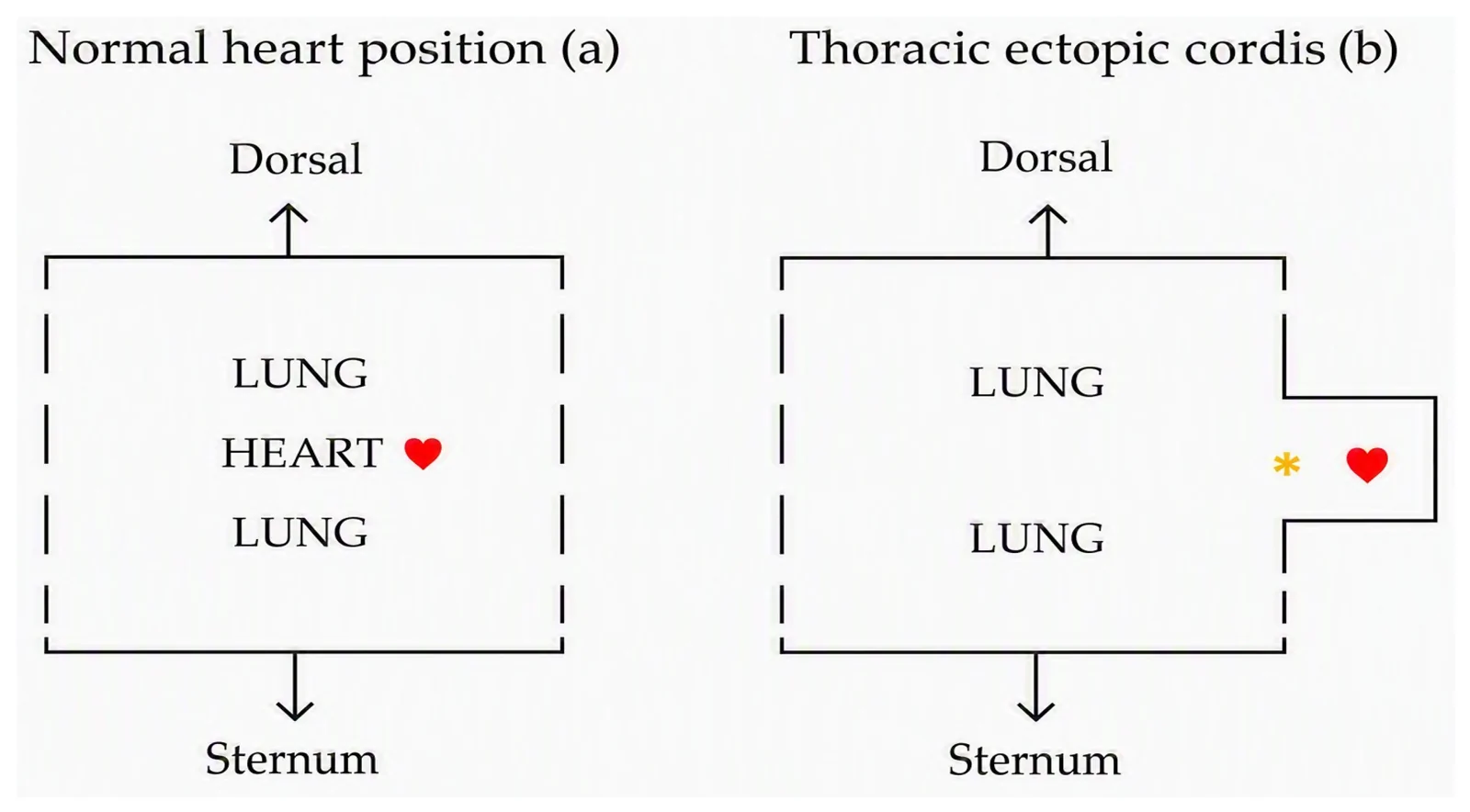
Schematic representation of the normal cardiac position (**a**) and thoracic ectopic cordis (**b**) in the calf. In the normal condition, the heart is enclosed within the thoracic cavity between the lungs and protected by the thoracic wall. In the affected calf, the heart was displaced subcutaneously through a thoracic cleft located at the level of the fifth and sixth intercostal right spaces (*).

At birth, the affected calf presented a subcutaneous ectopic heart visible in the right thoracic region (Supplementary Material—Video). The calf showed no obvious clinical impairment attributable to the congenital heart malformation (Figure 1). No cardiac ultrasonography evaluation was performed on the calf. The thoracic cavity and vital organs are shown in Figure 2. The presence of the pericardial sac can be observed, and rib segments were absent in the cardiac region of the thoracic wall.

### 2.2. Dissection

**Figure 3:**
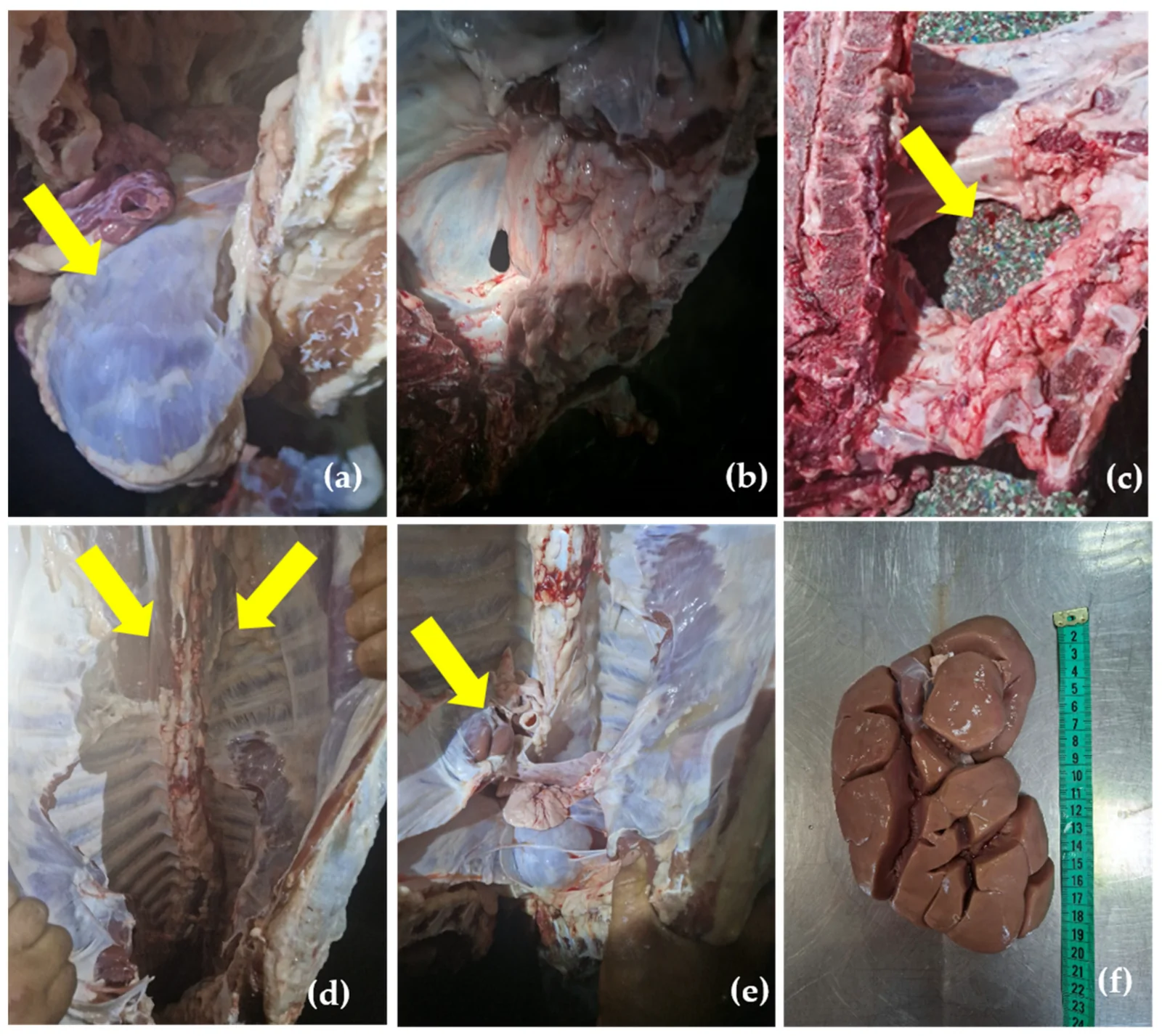
Postmortem findings in the anatomical regions of interest. Pericardium with the heart (yellow arrow) (**a**); right thoracic cleft (**b**,**c**); renal fossa of the kidneys (yellow arrow) (**d**); cranial displacement of the right kidney (**e**); and normal aspect of the right kidney (**f**).

**Figure 4:**
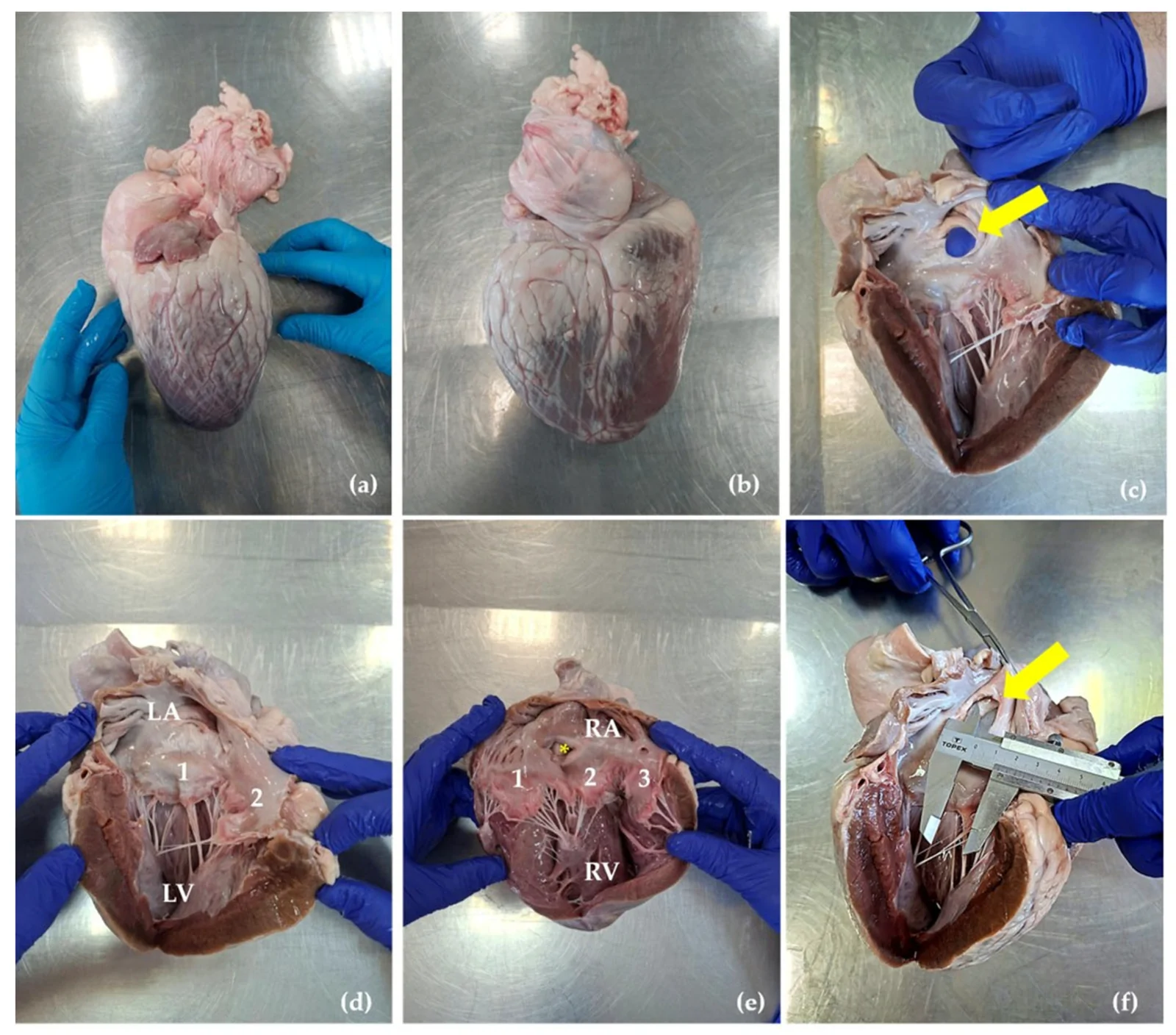
Macroscopic aspects of the heart: auricular face (**a**); atrial face with the pericardial sac (**b**); atrial septal defect (ASD) (yellow arrow) (**c**); left atrium (LA) and left ventricle (LV) with bicuspid valve (1—septal cusp, 2—parietal cusp) (**d**); right atrium (RA) and right ventricle (RV) with tricuspid valve (1—angular cusp, 2—septal cusp, 3—parietal cusp) and opening of the venae cavae (yellow asterix) (**e**); and measurement of ASD with the caliper (1.2 cm) (yellow arrow) (**f**).

At dissection, the heart was enclosed in the pericardial sac; the external conformation was normal (Figure 3). The superior diameter of the heart was 33 cm, the length along the long axis was 18 cm, and the heart weight was 950 g. In the proximal third of the interatrial septum, an atrial septal defect of 1.2 cm in diameter was observed (Figure 4). The right kidney appeared normal; its length was 19 cm, its width at the middle was 9.5 cm, and its weight was 550 g. (Figure 3).

### 2.3. Whole-Genome Sequencing Findings

Whole-genome sequencing was performed for two genomes, the affected male calf and his dam, to compare variants present in the calf with variants shared with or absent from the dam. The sire was not sampled; therefore, trio-based Mendelian inheritance testing was not possible. The alignment statistics revealed high mapping efficiency for both samples. In the CALF sample, 382,998,862 reads (89.20%) were uniquely mapped to the reference genome, while 42,304,959 reads (9.85%) were multi-mapped and 4,049,701 reads (0.94%) remained unmapped. Similarly, in the COW sample, 475,139,037 reads (90.43%) mapped uniquely, 45,980,297 reads (8.75%) were multi-mapped, and only 4,287,036 reads (0.82%) were unmapped. Additional read-quality and coverage metrics are summarized in Table 1. The consequences and zygosity of the 12 high-impact variants and the zygosity for the calf and for the cow are presented in Table 2. The VEP SIFT field classified 2270 missense variant annotations in the joint callset as deleterious. These SIFT-deleterious annotations are presented in Supplementary S1. Separately, VEP consequence annotation identified 12 high-impact start_lost variants (Table 3). Non-missense consequences such as start_lost and deletion were interpreted using VEP consequence and IMPACT annotations, not SIFT.

Analysis of genes implicated in cardiogenesis, as well as rib and renal development, identified the following genetic variants (Table 3).

Population allele-frequency annotation was not available for these candidate variants in the present analysis. Therefore, variants with existing rs identifiers, and variants also present in the dam, especially in a homozygous state, should be interpreted as standing bovine polymorphisms unless shown to be rare or absent in a suitable bovine reference database. “Predicted deleterious” refers only to in silico VEP/SIFT or VEP IMPACT annotation, while “candidate” refers to variants retained because of gene function and genotype pattern, and no variant is considered causal.

Exploratory gene annotations based on GO, KEGG and Reactome over-representation analyses are provided in Supplementary S2. These analyses were performed on the analyzed gene set after mapping gene symbols to Entrez identifiers for Bos taurus and Homo sapiens. The results should be interpreted as pathway-prioritization output and not as evidence of functional interaction among the candidate genes. The biological terms and pathways in the gene annotation databases consist of one KEGG pathway for *Bos taurus* and two KEGG pathways for *Homo sapiens*, one Reactome pathway for *Bos taurus* and three Reactome pathways for *Homo sapiens*.

KEGG pathway for *Bos taurus* identified the following term: Environmental Information Processing, signaling molecules and interaction category (BTA04512). KEGG pathway for *Homo sapiens* identified the same term (HSA04512), plus one term from the category organismal systems, subcategory sensory systems (HSA04740).

Reactome pathway annotations uncovered the following significant terms: Olfactory Signaling Pathway (R-BTA-381753) for *Bos taurus* and *Homo sapiens*, and expression and translocation of olfactory receptors (R-HAS-9752946) and ECM proteoglycans (R-HAS-3000178) for *Homo sapiens*. The summary of genetic variants identified by variant calling and joint genotyping is shown in Table 4 and Table 5.

Based on the genomic data from the calf and dam, the metrics show a high-quality WGS dataset, with some specific structural features such as a SNP-to-indel ratio of roughly 5.7:1, while the Ti/Tv ratio is 2.14. The SNP–indel ratio is expected for a multi-sample cohort, as indels are discovered more frequently as sample sizes increase. In cattle WGS studies, Ti/Tv ratio, the expected genome-wide transition-to-transversion ratio is typically 2.0–2.2. In our case, 2.14 indicates high-fidelity variant calling and low false-positive sequencing noise.

The high singleton rate in the private callsets suggests many variants are sample-specific within this two-sample comparison, but it does not replace external population allele-frequency filtering. A final candidate list should exclude variants with population frequency >1–5% in suitable bovine reference populations, unless there is a specific biological reason to retain a common variant.

Stratification of the Ts/Tv ratio by quality score revealed highly stable profiles across both datasets (Supplementary S3 file). The cumulative Ts/Tv ratios stabilized at 2.09 for CALF and 2.11 for COW, closely matching the expected ~2.1 baseline for true biological variants in the bovine genome. Evaluation of the local 1% bins (dashed lines) demonstrated a distinct, sharp decline in Ts/Tv values at the lower-quality tail of the distribution. Specifically, the local ratio fell sharply below 2.0 after approximately 1.2 sites for CALF and 1.3 sites for COW. This downward trajectory highlights an accumulation of sequencing errors and false positives within the lowest-quality tiers of the unfiltered private variants.

Mean per-base Phred quality and the percentage of reads with mean sequence quality >= Q30 were obtained from FastQC summaries. GC content was obtained from FastQC basic statistics. Mean chromosome-level depth, median coverage and the proportion of the genome covered at >=10x were obtained from mosdepth summaries.

## 3. Discussion

Congenital heart defects (CHDs) are cardiac diseases present at birth. In cattle, there has been a reported prevalence of 0.2% to 2.7–3%, but the actual prevalence may be underestimated because many calves may die during the perinatal period before veterinary examination or go undetected [15,16]. Ventricular septal defects (VSD) are reported to be the most common in ruminants; however, a vast collection has been reported (e.g., atrial septal defects (ASD), valvular hematomas, patent foramen ovale, and transposition of the aorta and pulmonary artery) [7,15,17]. They can also be subdivided into non-cyanotic anomalies (e.g., ASD, patent ductus arteriosus without pulmonary hypertension) and cyanotic (e.g., tetralogy of Fallot, Eisenmenger’s complex) [15]. CHD can occur as isolated defects or with syndromic evolution. In a 10-year retrospective study, 25% of all ruminants with CHD showed abnormalities of multiple organs [13]. In human medicine, CHDs are among the most common birth defects, accounting for approximately 8–11 per 1000 live births and 13.6% of live births with CHD have syndromic evolution. The etiology of CHD is complicated because, although it is known that there is a 90% hereditary risk, incomplete penetrance and variable expressivity mean that in only 20% of CHD cases the genetic cause is identified [18]. Pathological copy number variants, aberrant expression of lncRNAs, and upregulated or downregulated miRNAs have been described in the literature as being related to different types of CHDs [19]. Many of the discoveries from CHD transcriptome studies draw attention to biological pathways that concurrently open the door to a better understanding of cardiac development and related therapeutic avenues [20].

The VEP SIFT annotation identified 2270 deleterious missense variant annotations in the joint callset. In a separate VEP consequence-based analysis, 12 variants were classified as high-impact start_lost variants (Table 2). The genomic data show start-lost mutations in some olfactory receptor genes (*OR6K4*, *OR51H8*, and *OR8J17*). The OR gene families are the largest, highly polymorphic clusters in the mammalian genome [21], and pathway analysis is challenging due to very large gene families rather than true biological relevance [22]. The bovine genome harbors > 800 functional OR genes with high polymorphism [23]. Two other genes with predicted deleterious variants were *MBOAT4* and *MARCKS*; their pathogenic role in this case remains unproven. *MBOAT4* (membrane-bound O-acyltransferase domain-containing 4), or *GOAT* (ghrelin O-acyltransferase), primarily controls the post-translational modification of ghrelin and metabolic signaling for the ghrelin peptide to exert its physiological effects, such as growth hormone secretion and cardiovascular benefits [24].

Analyzing genes involved in cardiac and renal genesis, the following genes: *SHH*, *TBX18*, *NOTCH1*, *GATA3*, *TBX2*, *FOXC1*, and *RET* had mutations with moderate impact on the overlapped genes (Table 3). Among these genes, variant annotation focuses on genes known to be involved in cardiogenesis, rib development or renal development in mammals.

### 3.1. Variants in Genes Involved in Cardiac Morphogenesis

The development of the heart is a complex multistep process, like all other biological systems that form during the embryonic period. The stages are precardiac and cardiac mesoderm induction, cardiac crescent formation, heart tube formation, cardiac looping, and four-chambered fetal heart formation [25]. Numerous signaling pathways, such as transcription factors (e.g., *NKX2-5*, *GATA4*, etc.) and growth factors (BMP, FGF, etc.), Wnt, SHH, Notch signals and many others play pivotal roles in different phases of heart development, reflecting the complexity of the regulatory network involved in heart development [8,26,27].

The Hedgehog (Hh) signaling pathway, through the Sonic hedgehog (*SHH*) gene, is important for heart development. The importance of the *SHH* gene is highlighted by the cardiac defects observed in laboratory animals in situations of overexpression and downregulation of Hh pathway components [28,29]. Cardiogenic transcription factors, such as the *GATA* family, *Isl1*, *Tbx5*, *Nkx2.5*, *SRF*, and *HAND1*, are important in the transcription of genes related to heart formation [30,31].

Other genes, such as *TBX1*, *TBX2*, *TBX3*, *TBX5*, *TBX6*, *GATA4*, *NOS3*, *BMP2*, *Wnt*, etc., are important for the septation of the heart [32]. Members of the T-box gene family (Tbx) of transcription factors are important regulators of cardiac development. T-box genes (e.g., T*BX5*, *TBX1*, *TBX2*, *TBX3*, *TBX18*, and *TBX20*) are likely to contribute to cardiac lineage determination, chamber specification, valve formation, epicardial development or specialization of the conduction system [33]. The *TBX18* gene (T-box transcription factor 18) has been primarily implicated in the development of the kidney and urinary tract, where pathogenic variants have been associated with congenital anomalies [34]. As a member of the T-box transcription factor family, *TBX18* belongs to a group of genes that play crucial roles in embryonic development. Other T-box family members, including *TBX1* and *TBX5*, are well-established regulators of cardiac morphogenesis. In murine models, *TBX18* expression has been shown to play a critical role in the recruitment and differentiation of progenitor cells at the cardiac venous pole [35]. Homozygous *TBX18*−/− mice exhibit perinatal lethality and present with significant cardiac abnormalities [36,37]. In contrast, heterozygous *TBX18*+/− mice survive postnatally and do not display overt morphological cardiac defects [38]. Mutations in *TBX5* frequently lead to septal defects, while abnormalities in Notch and TGFβ signaling are often associated with defective valve development [25].

Notch signaling in neural-crest-derived cardiovascular tissues is required for aortic arch patterning and septation; neural crest-restricted Notch disruption in mice produces outflow, arch, and septal defects. This animal data strongly supports a CHD link, often in the outflow/AV canal spectrum that clusters with ASD/VSD [39].

In our case, a hedgehog-family gene, *SHH* (4:g. 117,486,610T>C), a Tbx family gene (*TBX18* gene with 9:g. 64,662,272delCTT), Notch pathway (*NOTCH1* gene with 3 mutations 11:g.103,935,934 G>T, 11:g.103,945,121 T>C and 11:g.103,952,273 T>C) or GATA family genes (*GATA3* gene 13:g.15,922,002 G>C) presented mutations. Thus, moderate-impact calls in *SHH*, *TBX18*, *NOTCH1* and *GATA3* occur in genes with known roles in cardiogenesis and may be biologically relevant to septation or outflow anomalies—biologically coherent with ASD and the body wall/ventral midline closure issues. However, the present data do not demonstrate statistical interaction, epistasis, or functional effects among these genes. In humans, the pathogenesis of CHD remains unclear, with only 15% of cases attributed to genetic inheritance and 30% associated with environmental risk factors, including fever, infections, maternal smoking, alcohol consumption, diabetes, and hypertension [40].

### 3.2. Variants in Genes Associated with Renal and Urinary Tract Development

*FOXC1* (Forkhead box C1) is a protein-coding gene that belongs to the forkhead family of transcription factors, which is characterized by a distinct DNA-binding forkhead domain. It plays a role in a broad range of cellular and developmental processes such as eye, bone, cardiovascular, kidney and skin development. Mutation of the *FOXC1* gene was associated with Axenfeld–Rieger syndrome in a Chinese family [41]. Human CAKUT (congenital anomalies of the kidneys and urinary tract) cohorts and mouse *FOXC1/FOXC2* studies show ureteric bud/urinary tract anomalies [8].

For the *FOXC1* gene (ENSBTAG00000064862), two mutations were identified, one is in the position 23:g.51,613,451 G>C (p.R406G) revealed a homozygous single nucleotide variant, both in calf and in the mother, and the consequence was a missense mutation, while the second mutation in the same gene was in the position 23:g.51,614,051delCGG (p.P205-) as a homozygous variant only in the dam’s genome.

The *RET* gene (ENSBTAG00000000570) from chromosome 28 had three variants; the first was located at position g.13,493,545 C>G (p.P161A), the other two were close to each other at genomic position g.13,501,854 A>G (p.I469M) and g.13,501,906 G>A (p.A487T). All three RET variants were missense variants, with g.13,501,854 A>G predicted as deleterious by the VEP SIFT annotation. *FOXC1* and *RET* are relevant to renal developmental pathways; however, because some variants were also present in the dam and population allele frequencies were not available, these findings should be considered provisional candidate observations rather than evidence of causality.

Joint variant calling identified 8,591,095 SNPs and 1,510,077 insertions/deletions across the analyzed genomes. The overall transition/transversion (Ts/Tv) ratio was 2.14, consistent with expectations for high-quality bovine whole-genome sequencing data. Approximately 33.2% of SNPs and 39.4% of indels were singletons, indicating the presence of a substantial proportion of rare variants. A total of 209,305 multiallelic sites were detected, including 12,227 multiallelic SNPs. The observed variant statistics and Ts/Tv values support the reliability and accuracy of the variant-calling pipeline.

The alignment of our cumulative Ts/Tv ratios with known mammalian genomic standards demonstrates the high stringency and accuracy of our variant-calling pipeline. While the private variant files contain a notable proportion of singletons, the steady Ts/Tv metrics within the high-quality tiers confirm that these rare, group-specific mutations reflect genuine biological diversity rather than technical artifacts. Moreover, the distinct inflection points observed in the stratified analysis provide an evidence base for defining hard-filtering thresholds, thereby ensuring that downstream analyses are performed only on high-confidence variant sites.

### 3.3. Biological Significance of the Identified Variants

Taken together, the identified variants occur in genes involved in developmental pathways that regulate cardiac morphogenesis, septation, outflow tract formation, and renal development. Several moderate-impact variants were shared by the calf and its dam, including variants in *TBX18*, *NOTCH1*, *GATA3*, *FOXC1*, and *RET*. The presence of these variants in the clinically unaffected dam argues against a direct causal effect of any single inherited variant. Although the detected variants were predicted to have only moderate impact and their functional consequences remain unknown, the results obtained contribute to knowledge of the genetic basis of congenital defects in cattle. The current study does not demonstrate a direct causal relationship between these variants and the congenital anomalies recorded.

The etiology of ectopia cordis is not known with certainty, but it is assumed to be complex, with a possible interaction between genetic and environmental factors [7]. It is assumed that the defect is mainly due to incomplete or defective closure of the embryonic lateral folds responsible for the ventral closure of the body wall. The ventral wall starts developing around the eighth day of embryogenesis when the mesoderm differentiates and grows, then moves laterally. These embryonic lateral folds form as a combination of the parietal layer of lateral plate mesoderm and overlying ectoderm and must move ventrally to meet in the midline. The fusion process between the folds is complex, involving cell-to-cell adhesion, cell migration, and cell reorganization. If there is an abnormal closure of the lateral body wall in the thoracic region, ectopia cordis can result ([42]).

Another possible mechanical cause for EC is the amniotic band syndrome, which is commonly caused by the premature rupture of the chorion or amnion, causing fibrous and sticky bands to interfere with various developing fetal parts, leading to EC or other deformities, such as midline sternal cleft, limb deformities, frontal-nasal dysgenesis [2,14].

The genetic factors that are associated with ectopic heart etiopathogenesis include mutations in genes that are involved in the formation and differentiation of cardiomyocytes, such as *BMP2* and *BMP4* [7,30]. These gene families, bone morphogenetic protein (BMP), together with *FGF8*, NODAL, and *Wnt*, represent the early molecular signaling for the formation of the future cardiac mesoderm during the pregastrula period. In this period, the interaction between the posterior epiblast and the hypoblast occurs to form the future cardiac mesoderm. The process continues with the migration of cardiac cells anterior to the primitive streak. From here, during gastrulation, cardiac cells migrate anterolaterally to form the left and right anterior lateral plate mesoderm. When the left and right precardiac mesoderm moves to the ventral midline and fuses, the single primitive heart tube is formed, and the heart starts to beat spontaneously [30,31]. The midline fusion and formation of the thoracic and abdominal cavities are completed by the ninth week of the embryonic period [14,42]. These mechanisms are similar in mammals, so cardiac morphogenesis described in humans can be attributed to cardiac development in animals [31].

Ectopia cordis has also been reported sporadically in chromosomal aneuploidies, such as mosaic trisomy 16 [43], trisomy 18 [44] or 46, XX, 17q+ [45]. Similarly, in veterinary medicine, a small marker chromosome was reported (XY, 61, mosaicism) in a calf with ectopia cordis [46]. Intrauterine drug exposure (calcium antagonist) in chicken embryo models has been linked to EC [47].

A final diagnosis should exclude the following differential congenital pathological conditions: cyllosomas, body stalk anomaly, Pentalogy of Cantrell, amniotic band syndrome. Cyllosomas is another congenital defect that can also involve ectopia cordis. Cyllosomas involves the presence of two of the following abnormalities: thoraco-abdominoschisis or abdominoschisis, limb defects or cranio-facial defects (cleft lip/palate, encephalocele, exencephaly, etc.) [3]. Also, in body stalk anomaly, ectopia cordis was observed, which is defined as a defect of embryonic blood flow in the phases of embryonic development, in weeks 4–6 of gestation. This condition results from a defect in the ventral wall’s closure and maintains the coelomic cavity’s persistence [48]. The Pentalogy of Cantrell (POC) is a pathology involving five congenital defects, including the thoraco-abdominal wall, sternal, diaphragmatic, pericardial and cardiac defects, such as ectopia cordis [11]. This syndrome has been reported in the veterinary specialty literature in a total of 19 animals (one calf, 10 dogs, two fish, six piglets) [11]. The embryo-pathogenesis proposed by Cantrell in this syndrome includes two developmental anomalies: the first, affecting the septum transversum and adjacent intraembryonic mesoderm, and the second refers to a failure of migration and fusion of the primordial sternum [11].

During dissection of the heart, an atrial septal defect (ASD) approximately 1 cm in diameter was observed. Because the atrial septal defect was small, the calf tolerated the defect well. During fetal life, communication (the foramen ovale) between the atria allows shunting of blood from the right atrium to the left atrium to bypass the uninflated lungs. In a newborn calf, an opening in the atrial septum is not a defect if the membrane covering the opening is approximately 75% around the circumference. The normal membrane prevents left-to-right blood flow. Between 7 and 14 days of age, the membrane becomes more opaque, white in color, thicker and fuses completely with the atrial septum [49].

An important review explored the genetic basis of congenital cardiac septal defects and focused on key genes (e.g., *TBX5*, *TBX20*, *NKX2.5*, *GATA4*, *MYH6*, *CITED2*). These genes regulate critical processes in heart development, including septation, chamber formation, and myocardial differentiation. Mutations or variations in these genes can lead to deficiencies in cardiac structure and function, resulting in CHDs [32]. Treatment of large interatrial communications in dogs by open-heart surgery has been reported [50], as has closure using expanded polytetrafluoroethylene, where the arrhythmias postoperatively were temporary [51].

Another anomaly identified in this case was unilateral renal agenesis. This birth defect is caused by a failure of kidney formation during fetal development. If the contralateral organ compensates adequately, the animal can be asymptomatic. Dissection of the right kidney did not identify any compensatory hypertrophy, probably due to the short life of the calf. No abnormalities of the reproductive tract were observed in the affected calf.

The development of the kidney takes place due to interactions between the ureteric bud of the mesonephric duct and the metanephric mesenchyme. Renal agenesis occurs when the ureteric bud fails to form the ureter, the renal pelvis and the collecting ducts [52]. The other congenital pathology is renal dysplasia due to congenital absence or defective development of the metanephric mesenchyme to form the kidney.

The etiology of this pathology is different, so if during embryonic development the ureteric bud is not formed, we are talking about renal agenesis, and if the metanephric mesenchyme is defective or poorly developed, the diagnosis is renal aplasia.

Mutations in *GDNF* (glial-cell derived neurotrophic factor) and *RET*, which play a key role in the induction of the ureteric bud, have been attributed to renal agenesis in humans [53,54,55], whereas mutations in *FGF20*, *GREB1L*, and *DSTYK* are responsible for renal hypoplasia/aplasia [56]. Renal agenesis was reported in dogs (OMIA 002007-9615), domestic cats (OMIA 002007-9685) and rabbits (OMIA 002007-9986).

Each case of congenital anomalies with syndromic evolution is almost unique. An example is an apparently functional Friesian calf with tetralogy of Fallot, double outlet right ventricle (both the aorta and the pulmonary artery emerged from the right ventricle), patent foramen ovale and persistent right aortic arch. Like the case described by us, the calf also had agenesis of the left kidney [57]. This finding reinforces the importance of a thorough examination for coexisting congenital malformations in animals affected by ectopic cordis.

## 4. Materials and Methods

### 4.1. Ethics Statement

The case study was performed in compliance with national (Romanian Law 43/2014) and European guidelines (EU Directive 63/2010) for using animals for scientific purposes, and in accordance with the internal SOP (Standard Operating Procedures) of USAMVBT-PG-001-R021 (Banat’s University of Agricultural Sciences and Veterinary Medicine). Ethical approval was not required for this study because no experiments were conducted on live animals.

### 4.2. Animal

A male Romanian Spotted calf weighing 45 kg was born alive after a normal gestation period in May 2024, in a village in Northwestern Romania. Its mother was a Romanian Spotted cow in her 4th gestation, and the sire was a Romanian Spotted bull. The mother had no other calves with congenital abnormalities. The cow was bred by artificial insemination. Blood samples were collected from the jugular veins of both the calf and its dam into EDTA-containing vacutainer tubes by a veterinarian and subsequently submitted to the Faculty of Veterinary Medicine under appropriate transport and storage conditions.

The health of the calf was not affected by the visible congenital malformation after birth, so it was raised by its owners until 6 months of age, when it was slaughtered by the owner outside the scope of the study. The ectopic heart and right kidney were submitted to the Faculty of Veterinary Medicine from Timisoara, Genetics discipline, for examination.

### 4.3. Whole-Genome Sequencing

Genomic DNA of the calf and his mother was isolated from whole blood using a DNeasy Blood and Tissue Kit (Qiagen, Hilden, Germany) for DNA extraction. Whole-genome sequencing was performed by Novogene, Cambridge, United Kingdom. The calf sample had a volume of 35 µL and a concentration of 70 ng/µL DNA, while the mother sample had a volume of 30 µL and a concentration of 40 ng/µL DNA. The DNA concentration was measured using a Qubit^®^ DNA Assay Kit on a Qubit^®^ 2.0 Fluorometer (Life Technologies, Carlsbad, CA, USA).

Next, the library preparation was performed. Briefly, the genomic DNA was randomly fragmented into 350 bp fragments, and then the DNA fragments were end-polished, A-tailed, and ligated with the adapters for Illumina sequencing, and further PCR-enriched with P5 and P7 oligo primers. The final PCR products of the libraries were purified (AMPure XP system), followed by a quality-control test that included the size distribution using an Agilent 2100 Bioanalyzer (Agilent Technologies, Santa Clara, CA, USA) and the measurement of molarity using real-time PCR.

Raw sequenced reads in FASTQ format were analyzed using the nf-core/sarek pipeline v. 3.5.1, using Nextflow v. 25.04.8. The quality control of the raw data was performed with FastQC v. 0.12.1 [58] and summarized with MultiQC. Alignment and variant-processing outputs were generated with BWA-MEM v. 0.7.18, samtools v. 1.21, GATK v. 4.5.0.0, mosdepth v. 0.3.8, bcftools stats v. 1.2, vcftools v. 0.1.16, and Ensembl VEP v. 113.0. The FASTA file with genomic sequences and the corresponding GTF file with genomic locations for the ARS-UCD1.2 reference were acquired from Ensembl, release 115 [59]. The variant calling was performed with GATK HaplotypeCaller v. 4.5.0.0 [60]. Low-complexity regions consisting of microsatellites and simple repeats were downloaded from UCSC for the ARS-UCD1.2 reference and excluded from the variant-calling process. Variant effects on gene transcripts were predicted using the Ensembl Variant Effect Predictor (VEP) tool v. 113.0 [61] and the Ensembl GTF file previously mentioned.

Read-level QC metrics, including GC content, mean per-base Phred quality, and the percentage of reads with mean sequence quality >= Q30, were obtained from FastQC summaries compiled by MultiQC. Coverage metrics, including mean chromosome-level depth, median coverage, and the proportion of the genome covered at >=10x, were obtained from mosdepth summaries compiled by MultiQC.

The variants classified as having a high or moderate impact and the corresponding genes were retained for the downstream analysis, which was performed using the R programming language v. 4.3.3 [62]. Variant effects on transcripts were annotated with VEP, VEP consequence, IMPACT, and SYMBOL. Existing variation and SIFT fields were extracted with bcftools +split-vep and analyzed in R v. 4.3.3 with VariantAnnotation and vcfR. Candidate filtering was based on VEP IMPACT, the gene set selected for cardiac, thoracic wall or renal relevance, and genotype state in the calf and dam. High-impact variants were defined by VEP IMPACT == HIGH. Moderate-impact variants were defined by VEP IMPACT == MODERATE. SIFT was used only for missense variants, and missense variants were considered SIFT-deleterious only when the VEP SIFT field reported deleterious, corresponding to a SIFT score < 0.05. Start lost, deletion and other non-missense consequences were interpreted as VEP consequence classes and were not labeled as SIFT-deleterious. Genotypes were compared by splitting GT strings on “/” or “|” and checking whether a non-reference allele was present in the calf, in the dam, in both, or in the dam only. Because the sire was not sequenced, formal trio-based Mendelian inheritance testing and confirmation of de novo status were not possible.

Finally, gene annotations were obtained via the AnnotationHub package v. 3.10.0 [63] and multiple over-representation analyses based on GeneOntology (GO) [64], Kyoto Encyclopedia of Genes and Genomes (KEGG) [65], Medical Subject Headings (MeSH) [66], and Reactome [67] were performed with the R packages clusterProfiler v. 4.10.0 [68], meshes v. 1.28.0 [69], and ReactomePA v. 1.46.0 [70] using enrichPathway for organism = “bovine” and organism = “human” with pvalueCutoff = 0.05. Disease ontology analysis was performed with DOSE using enrichDO with ont = “DO”, pvalueCutoff = 0.05, pAdjustMethod = “BH,” and readable = TRUE. In order to perform the previously mentioned over-representation analyses, common gene identifiers reported by Ensembl VEP for high-impact and moderate-impact variants were searched within the corresponding OrgDb and MeSHDb annotation databases from AnnotationHub for both *Homo sapiens* and *Bos taurus*, using the *select* function from the AnnotationDbi package v. 1.64.1 [71]. Gene annotation was performed on sets of all genes associated with high-impact and/or moderate-impact variants, in order to find significantly associated annotation terms with the specific variants found in this study.

The raw sequencing data have been uploaded to the NCBI SRA database under the accession number PRJNA1496329.

To assess the quality and reliability of the discovered single-nucleotide polymorphisms (SNPs) in the private callsets (CALF. and COW.p), the transition-to-transversion (Ts/Tv) ratio was evaluated as a function of variant quality score (QUAL). Variants were ranked by QUAL score in descending order, and cumulative and binned (1%) Ts/Tv ratios were computed to identify the inflection points where sequencing artifacts and false positives begin to dominate the dataset.

## 5. Conclusions

This case combines thoracic ectopia cordis, atrial septal defect, and unilateral renal agenesis in a Romanian Spotted calf. Whole-genome sequencing of the calf and dam identified VEP high-impact variants and moderate-impact candidate variants in genes with reported roles in cardiac, thoracic wall, or renal development. These findings are exploratory and do not establish a causal variant or a polygenic interaction, but the results obtained contribute to knowledge of the genetic basis of congenital defects in cattle. Population allele-frequency annotation, sequencing of the sire and additional relatives, segregation analysis in related animals, and functional validation will be required to determine whether any of the reported candidates contribute to the phenotype.

## Figures and Tables

**Table 1:** Additional whole-genome sequencing quality-control metrics for the calf and dam.

Metric	CALF	COW
Total read pairs	214,676,761	262,703,185
Total reads	429,353,522	525,406,370
Reads mapped (%)	99.06	99.18
Mean per-base Phred quality	39.01	39.05
Reads with mean sequence quality >= Q30 (%)	98.48	98.46
GC content (%)	44.0	44.0
Mean chromosome-level depth (x)	16.41	20.54
Median coverage (x)	16	20
Genome covered at >=10x (%)	95.0	99.0

**Table 2:** VEP high-impact start_lost variants identified in the joint callset.

Gene	Type of Variant	Variant ^a^	Existing Variant ID	Consequence of the Variant	Zygosity in Calf ^b^	Zygosity in Cow ^c^
ENSBTAG00000051403	substitution	2:g. 77,262,461 T>C	rs209860758	start_lost	HET	HET
*OR6K4*	substitution	3:g. 10,946,349 G>T	rs109473950	start_lost	-	HET
ENSBTAG00000040331	substitution	4:g. 113,133,654 C>T	rs453954220	start_lost	-	HET
ENSBTAG00000008550	substitution	4:g. 113,285,716 T>C	rs472654149	start_lost	HET	HET
ENSBTAG00000008550	substitution	4:g. 113,285,717 G>A	rs446868044	start_lost	HET	HET
*MARCKS*	substitution	9:g. 36,558,112 T>A	rs480782291	start_lost	HET	-
ENSBTAG00000056248	substitution	9:g. 87,265,583 T>G	NA	start_lost	HET	HET
*OR51H8*	substitution	15:g. 48,852,452 A>G	rs383601178	start_lost	HET	HOM
*OR51F5C*	substitution	15:g. 50,261,811 A>G	rs109495325	start_lost	HOM	HET
*OR8J17*	substitution	15:g. 79,607,878 G>A	rs377869031	start_lost	HOM	HET
ENSBTAG00000051483	substitution	17:g. 70,798,284 A>G	NA	start_lost	HET	HET
*MBOAT4*	substitution	27:g. 26,315,496 A>G	rs110991645	start_lost	HET	-

^a^ Given positions correspond to chromosomes of the ARS-UCD1.2 assembly reference bovine genome. ^b,c^ HOM—homozygous, HET—heterozygous, NA—not applicable. A dash in the zygosity column indicates that there is no mutation, i.e., homozygous reference.

**Table 3:** VEP moderate-impact variants in genes selected for relevance to cardiogenesis, rib development, or renal development.

Gene	Type of Variant	Variant ^a^	Existing Variant ID	Consequence of the Variant	Zygosity in Calf ^b^	Zygosity in Cow ^c^
*SHH*	substitution	4:g.117,486,610T>C	rs379854710	missense	HET	-
*TBX18*	deletion	9:g.64,662,272delCTT	rs384279354	deletion	HET	HET
*NOTCH1*	substitution	11:g.103,935,934 G>T	rs383080834	missense	HET	HET
*NOTCH1*	substitution	11:g.103,945,121 T>C	rs110815092	missense	HET	HET
*NOTCH1*	substitution	11:g.103,952,273 T>C	rs109811642	missense	HET	HET
*GATA3*	substitution	13:g.15,922,002 G>C	rs42309884	missense	HET	HET
*TBX2*	substitution	19:g.11,686,948 C>A	rs377969988	missense	HET	-
*FOXC1*	substitution	23:g.51,613,451 G>C	rs210973470	missense	HOM	HOM
*FOXC1*	deletion	23:g.51,614,051delCGG	rs5383072260	deletion	-	HOM
*RET*	substitution	28:g.13,493,545 C>G	rs110056366	missense	HOM	HET
*RET*	substitution	28:g.13,501,854 A>G	rs110630023	missense	HOM	HET
*RET*	substitution	28:g.13,501,906 G>A	rs383954348	missense	HET	-

^a^ Given positions correspond to chromosomes of the ARS-UCD1.2 assembly reference bovine genome. ^b,c^ HOM—homozygous, HET—heterozygous. The line in the zygosity represents that there is no mutation; reference homozygous.

**Table 4:** The summary of genetic variants identified by variant calling and joint genotyping.

	**SNPs**			**Indels**		**MNPs**	**Others**
**Callset**	**n**	**ts/tv**	**(1st ALT)**	**n**	**frm** *		
Joint	8,591,095	2.14	2.15	1,510,077	-	0	0
	**Singletons (AC = 1)**			**Multiallelic**			
**Callset**	**SNPs**	**ts/tv**	**Indels**	**Sites**		**SNPs**	
Joint	33.2%	2.08	39.04%	209.305		12.227	

* frameshift ratio: out/(out + in)

**Table 5:** Summary statistics of genetic variants identified in the CALF and COW datasets (private.vcf.gz).

	**SNPs**			**Indels**		**MNPs**	**Others**
**Callset**	**n**	**ts/tv**	**(1^st^ ALT)**	**n**	frm *	**0**	**0**
**CALF**	1,337,521	2.09	2.09	221,802	-	0	0
**COW**	1,569,309	2.10	2.11	283,469	-	0	0
**CALF + COW**	0	0.00	0.00	0	-	0	0
		**Singletons (AC = 1)**		**Multiallelic**			
**Callset**	**SNPs**	**ts/tv**	**Indels**	**Sites**		**SNPs**	
**CALF**	97.2%	2.10	93.8%	3367		268	
**COW**	97.3%	2.12	94.2%	5426		112	
**CALF + COW**	0.0%	0.00	0.0%	0		0	

* frameshift ratio: out/(out + in)

## Data Availability

The original data presented in the study are openly available in the NCBI SRA under accession number PRJNA1496329.

## Supplementary Materials

The following supporting information can be downloaded at: https://www.mdpi.com/article/10.3390/ijms27188179/s1.
