# Supplementary material for: The First Genomic Study in a Romanian Spotted Calf with Ectopia Cordis Thoracalis
Source: Int J Mol Sci. 2026 Sep 14;27(18):8179. doi: 10.3390/ijms27188179 (PMC13607540; doi:10.3390/ijms27188179)

Total counts

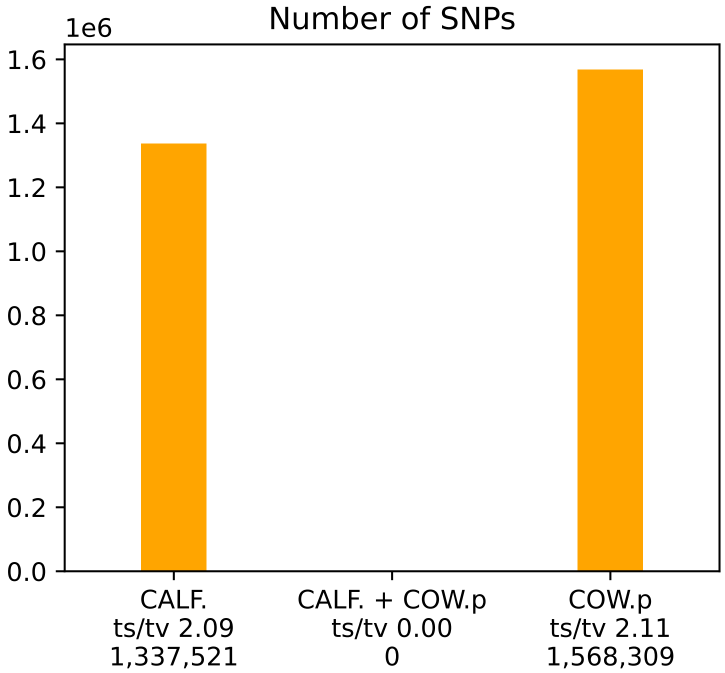

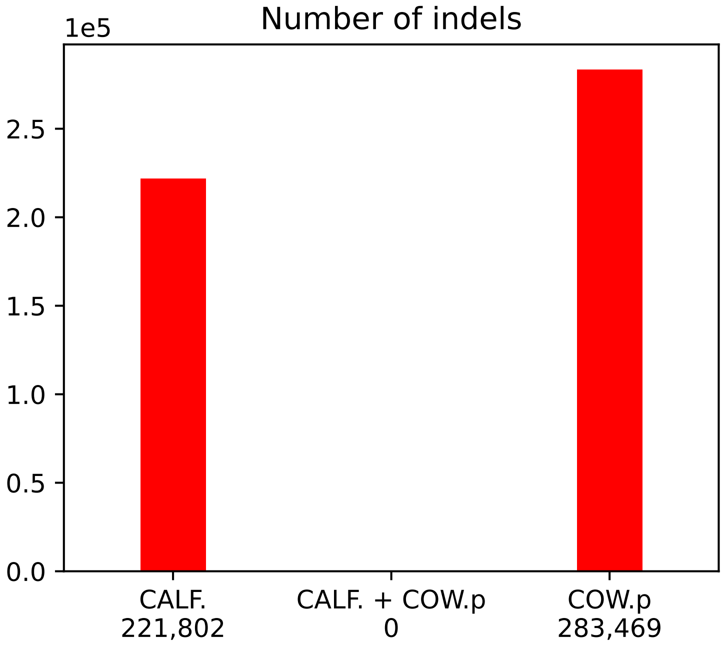

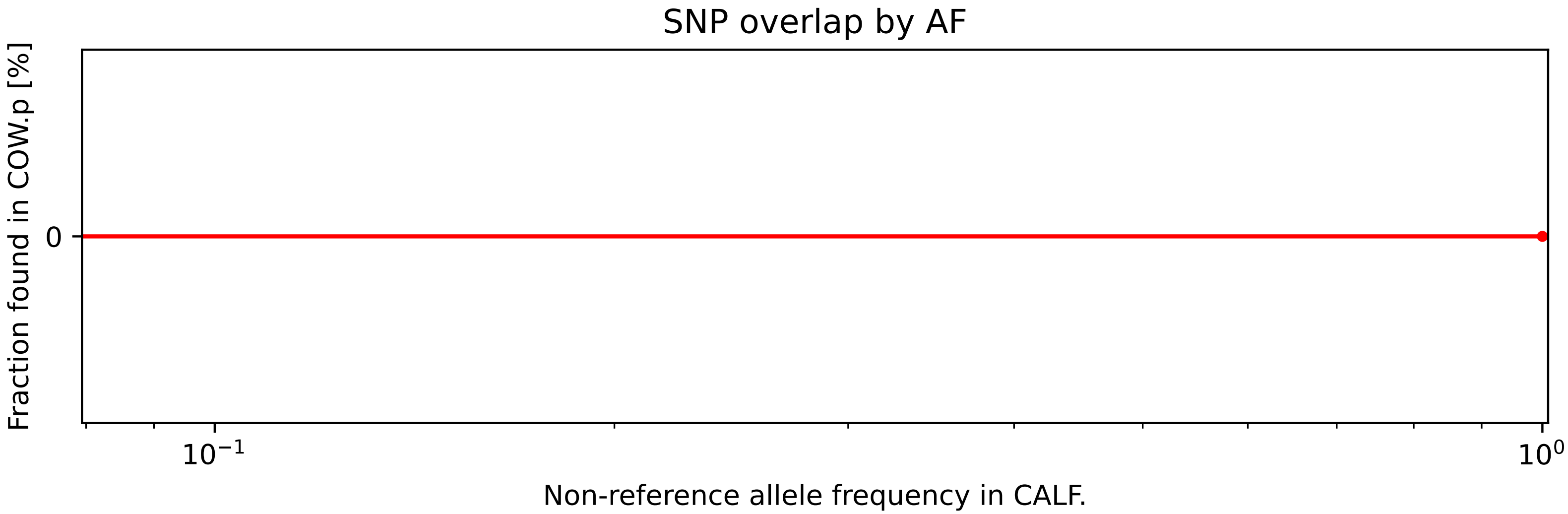
SNP overlap by AF
Fraction found in COW.p [%]
0
10^{-1}
10^{0}
Non-reference allele frequency in CALF.

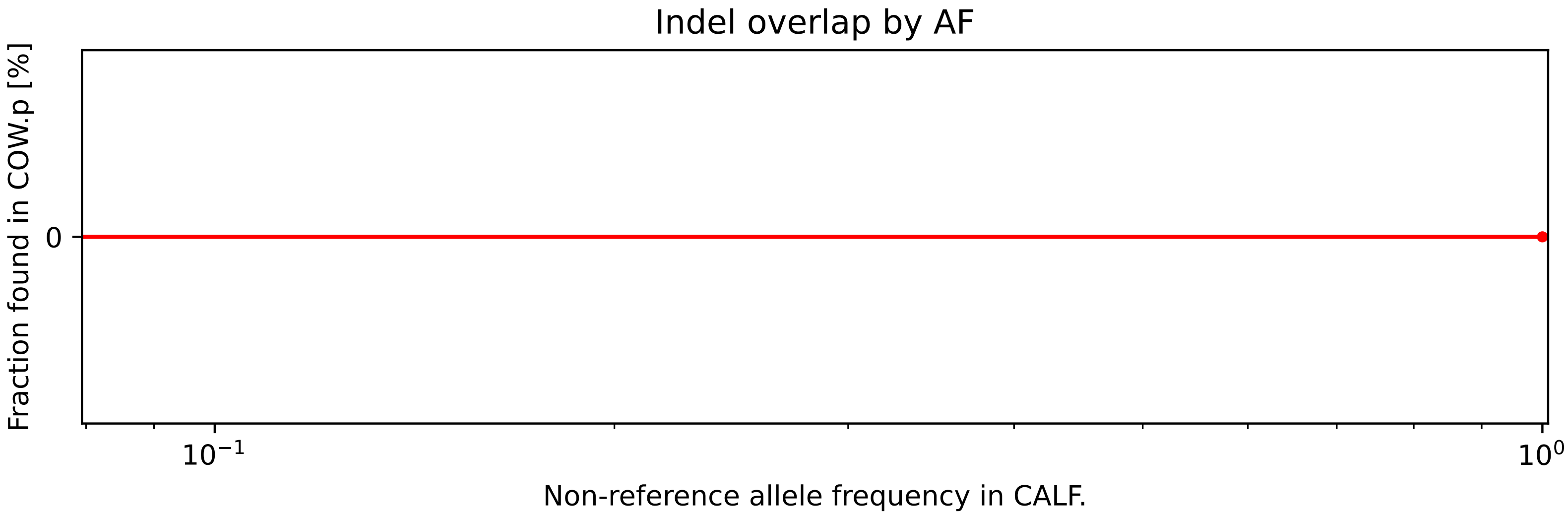
Indel overlap by AF
Fraction found in COW.p [%]
0
10^{-1}
10^{0}
Non-reference allele frequency in CALF.

Ts/Tv stratified by QUAL

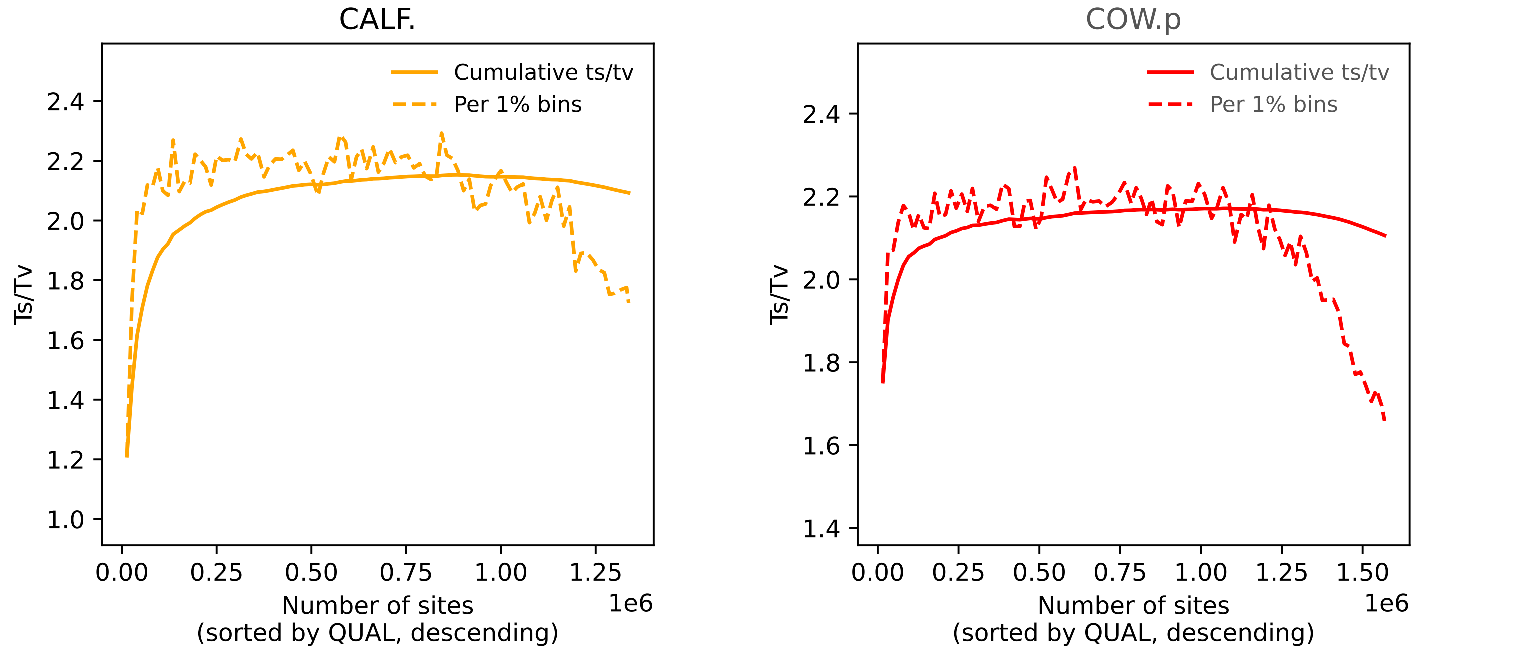

Indel distribution

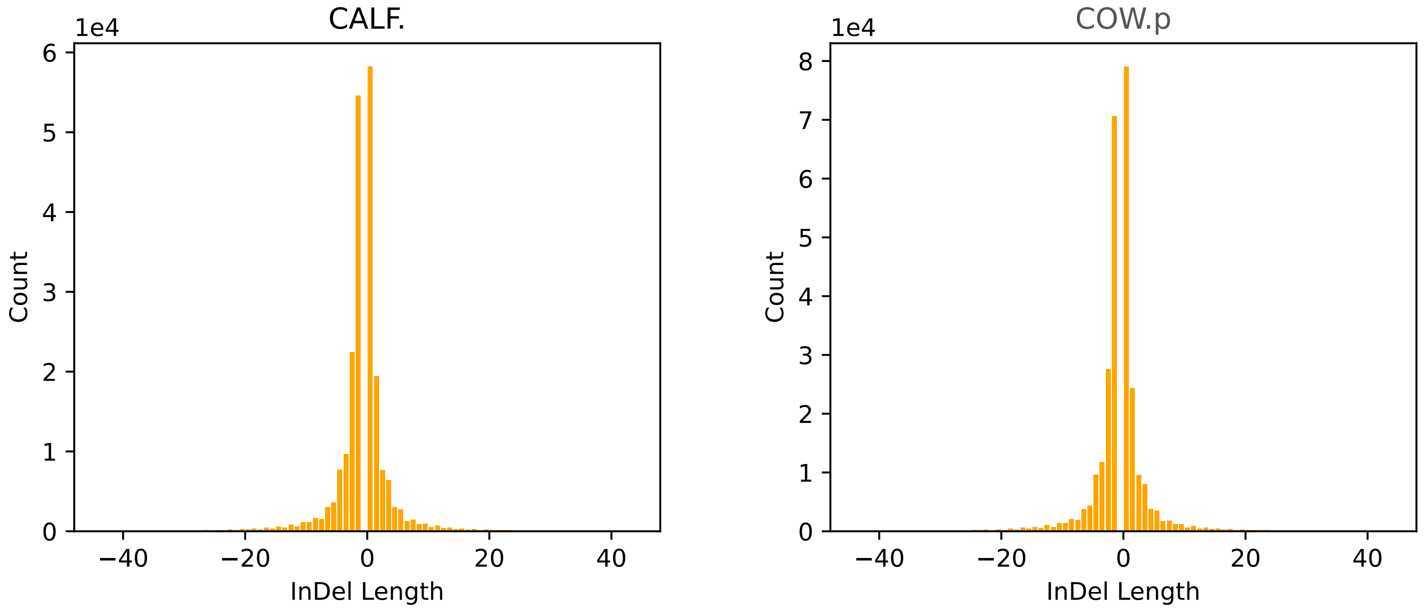

Depth distribution

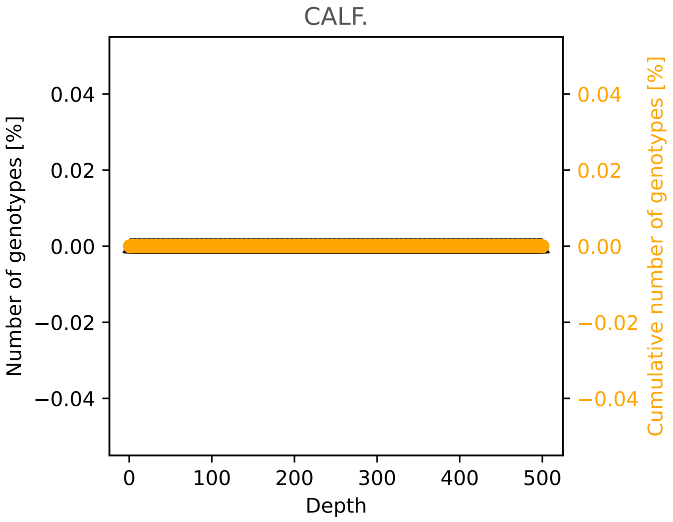

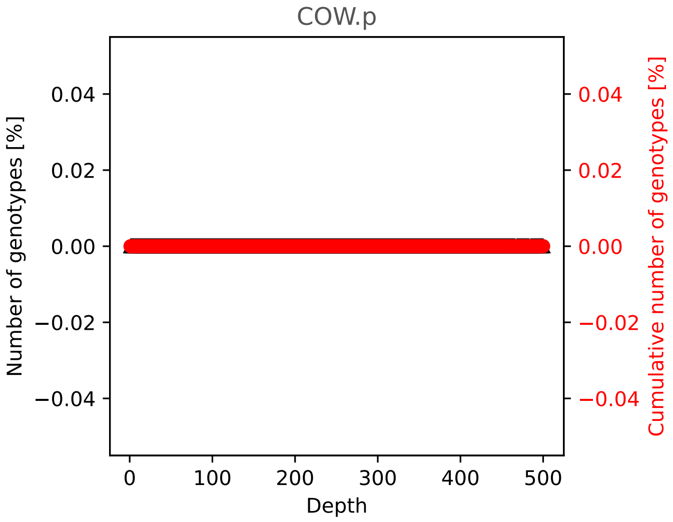

Substitution types

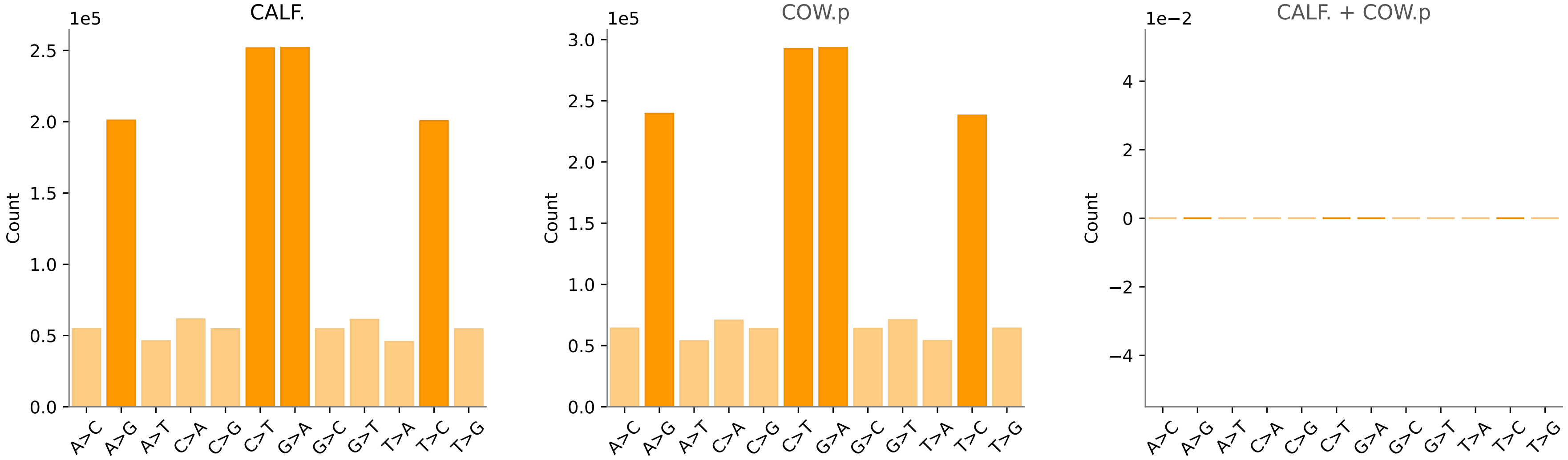

Supplement: Supplementary file 1 [file ijms-27-08179-s001.zip › ijms-4490046 Supplementary S3 file.pdf]
